# Assessing metabolic syndrome prediction quality using seven anthropometric indices among Jordanian adults: a cross-sectional study

**DOI:** 10.1038/s41598-022-25005-8

**Published:** 2022-12-06

**Authors:** Islam Al-Shami, Hana Alkhalidy, Khadeejah Alnaser, Tareq L. Mukattash, Huda Al Hourani, Tamara Alzboun, Aliaa Orabi, Dongmin Liu

**Affiliations:** 1 Department of Clinical Nutrition and Dietetics, Faculty of Applied Medical Sciences, The Hashemite University, Zarqa, 13133 Jordan; 2 Department of Nutrition and Food Technology, Faculty of Agriculture, Jordan University of Science and Technology, Irbid, 22110 Jordan; 3 Department of Clinical Pharmacy, Faculty of Pharmacy, Jordan University of Science and Technology, Irbid, 22110 Jordan; 4 Department of Human Nutrition, Foods and Exercise, College of Agriculture and Life Sciences, Virginia Tech, Blacksburg, VA 24061 USA

**Keywords:** Obesity, Body mass index

## Abstract

Metabolic syndrome (MSyn) is a considerable health concern in developing and developed countries, and it is a critical predictor of all-cause mortality. Obesity, specifically central obesity, is highly associated with MSyn incidence and development. In this study, seven anthropometric indices (Body Mass Index (BMI), Waist circumference (WC), Waist-to-Height Ratio (WHtR), A Body Shape Index (ABSI), Body Roundness Index (BRI), conicity index (CI), and the Visceral Adiposity Index (VAI)) were used to identify individuals with MSyn among the Jordanian population. These indices were assessed to identify their superiority in predicting the risk of MSyn. A total of 756 subjects (410 were male and 346 were female) were met between May 2018 and September 2019 and enrolled in this study. Height, weight, and waist circumferences were measured and BMI, WHtR, ABSI, BRI, CI, and VAI were calculated. Fasting plasma glucose level, lipid profile, and blood pressure were measured. Receiver-operating characteristic (ROC) curve was used to determine the discriminatory power of the anthropometric indices as classifiers for MSyn presence using the Third Adult Treatment Panel III (ATP III) definition. MSyn prevalence was 42.5%, and obese women and men have a significantly higher prevalence. BRI and WHtR showed the highest ability to predict MSyn (AUC = 0.83 for both indices). The optimal cutoff point for an early diagnosis of MSyn was > 28.4 kg/m^2^ for BMI, > 98.5 cm for WC, > 5.13 for BRI, > 0.09 m^11/6^ kg^−2/3^ for ABSI, > 5.55 cm^2^ for AVI, > 1.33 m^3/2^ kg^−1/2^ for CI, and > 0.59 for WHtR with males having higher cutoff points for MSyn early detection than females. In conclusion, we found that WHtR and BRI may be the best-suggested indices for MSyn prediction among Jordanian adults. These indices are affordable and might result in better early detection for MSyn and thereby may be helpful in the prevention of MSyn and its complications.

## Introduction

Metabolic syndrome (MSyn) is a cluster of several metabolic disorders that are significantly related to raising the risk for the development of cardiovascular diseases (CVD) and type 2 diabetes mellitus (Type 2 DM)^1^. MSyn is considered a critical predictor of all-cause mortality and premature mortality from cardiovascular events^2^. MSyn has become a considerable health concern in developing and developed countries^3^. The prevalence of MSyn worldwide varies widely depending on ethnicity and the criteria applied to define it^4^. Misra and Khurana^5^ reported that the prevalence of MSyn in low and middle-income countries ranges from 10 to 47%. The prevalence of MSyn among Jordanian adults ranged from about 36% to more than 51%, according to the National Cholesterol Education Program (NCEP), Third Adult Treatment Panel III (ATP III) criteria^6,7^.

There was a solid and direct association between MSyn and obesity, specifically central obesity; that is considered one of the MSyn components in different criteria sets. Recent studies showed evidence supporting the hypothesis that abdominal visceral fat plays a role in the development of MSyn^8^. A growing body of epidemiologic evidence shows that simple and inexpensive anthropometric measures can be used to predict MSyn^9^. These include measurements such as body mass index (BMI) and waist circumference (WC), which have been used in clinical practice for decades, as well as novel measures such as waist-to-height ratio (WHtR)^10–12^, A Body Shape Index (ABSI)^13–15^, the Body Roundness Index (BRI)^16,17^, the conicity index (CI)^18–20^, and the Visceral Adiposity Index (VAI)^21,22^.

Limitations of the old and routine indicators such as BMI and WC lead to continuous searching for new and more applicable methods to predict and diagnose MSyn. The BMI has been criticized because it does not discern between fat mass and muscle mass or reflect an individual's fat distribution^23^. On the other hand, WC is used to diagnose abdominal obesity, but it is highly correlated with BMI and is affected by body size (weight and height)^24^. Although some studies presented WC and BMI as best predictors for MSyn^25^ or the best tools for MSyn identification in a specific age or gender groups^26^; others found that using the newly recommended indices might result in a better prediction. For instance, a previous study showed that some anthropometric indices, including the lipid accumulation product and the cardiometabolic index were better predictors for MSyn^27^. Also, another study presented WHtR as an alternative obesity index that can modulate BMI limitations. Also, some previous studies suggested the WHtR is a superior indicator for cardiometabolic risk factors, CVD, risks for mortality, and MSyn when compared to BMI^28,29^. Another anthropometric indicator, ABSI, has been considered an independent index of height, weight, and BMI that predicts premature mortality better than BMI^13^. In 2013, Thomas and colleagues^16^ developed the BRI, which is based on WC and height, and it was displayed as a good indicator of body fat. Some recent studies defined the BRI as the anthropometric index with a superior ability to predict MSyn than other indices^30,31^. As a result of the assumption that body shape changes with abdominal fat accumulation, Valdez^19^ formulated an equation that evaluates WC in relation to weight and height and expresses it as the Conicity Index (CI). In 2019, Quaye and colleagues^32^ better predicted MSyn and its components using CI. Another anthropometric index is the VAI, which is based on two anthropometric indices (WC and BMI) and two biochemical factors (triglyceride (TG) and high-density lipoprotein (HDL)) and has a separate formula for men and women^21^. Although VAI might not be very cost-beneficial, Baveicy and others found that VAI is a better predictor of MSyn than BRI^33^.

Only a few studies have been conducted using some of these indices, and none compared the prediction qualities of seven anthropometric indices among Jordanian adults. Early identification of at-risk individuals is crucial as it will facilitate the designing of MSyn risk factors modification programs and prevent the onset and progression of MSyn later in life. Developing an effective intervention to manage and prevent the development of MSyn will finally result in controlling the development of CVD and Type 2 DM, or better than that, it can play a significant role in the prevention of the leading causes of mortality. So, this study aims were to assess the capacity of the seven anthropometric indices (BMI, WC, WHtR, ABSI, BRI, CI, and VAI) to identify individuals with MSyn among the Jordanian population. Moreover, to compare the attributes of recent anthropometric measurements (ABSI, BRI, CI, and VAI) and attempted to determine whether they were superior to BMI, WC, and WHtR. As well as, to identify the optimal cutoff points for the seven indices for predicting the risk of MSyn and offering appropriate preventive interventions in Jordan.

## Results

### Baseline characteristics of the participants

A total of 756 Jordanian adults participated in this study, of whom 410 (54.2%) were males, and 346 (45.8%) were females. The general characteristics are shown in (Table 1). According to the study's baseline data, there was no significant difference in age categories and marital status between both genders (*p* > 0.05). The results showed that more men had an education level less than a bachelor's degree (72.7% vs. 63.9%; *P* = 0.009) and were current smokers (51% vs. 11.3%; *p* < 0.001) compared to women.

**Table 1:** Characteristics of the study population stratified by gender.

Variable	Total N (%)	Males (n = 410)	Females (n = 346)	*P*-value
**Age (years)**				
20–34	244 (32.3)	140 (34.1)	104 (30.1)	0.061
35–44	219 (29)	124 (30.2)	95 (27.5)	
45–54	178 (23.5)	98 (23.9)	80 (23.1)	
55–65	82 (10.8)	33 (8)	49 (14.2)	
≥ 65	33 (4.4)	15 (3.7)	18 (5.2)	
**Marital status**				
Married	599 (79.2)	325 (79.3)	274 (79.2)	0.979
Single	157 (20.8)	85 (20.7)	72 (20.8)	
**Education**				
< B. Sc	519 (68.7)	298 (72.7)	221 (63.9)	**0.009**
≥ B. Sc	237 (31.3)	112 (27.3)	125 (36.1)	
**Cigarette smoking status**				
Non-smoker	210 (27.8)	92 (22.4)	118 (34.1)	** < 0.001**
Current smoker	248 (32.8)	209 (51)	39 (11.3)	
Former and Negative smoker	298 (39.4)	109 (26.6)	189 (54.6)	
**Family income level (Jordanian Dinars)**				
< 350 JODs	245 (32.4)	117 (28.5)	128 (37)	**0.047**
350—575 JODs	383 (50.7)	220 (53.7)	163 (47.1)	
800 or more	128 (16.9)	73 (17.8)	55 (15.9)	

Data are presented as Numbers and Percentages (N (%)).

Chi-square test was performed to test the differences between genders.

*B.Sc.* Bachelor's degree.

*P < 0.05 in the comparison between Males and Females.

Significant values are in bold.

### Prevalence of MSyn and its individual components

The results showed that MSyn was found in 42.5% of the study population; 38.5% in males and 47.1% in females (*p* = 0.018), according to the ATP III criteria. About a quarter of the study population (26.5%) had two metabolic abnormalities, 19.3% had one metabolic abnormality, and only 11.8% did not have any metabolic abnormality (Table 2).

**Table 2:** The prevalence of metabolic syndrome (MSyn) and its individual metabolic abnormalities stratified by gender.

Variable	Total N (%)	Males	Females	*P-*value
**Waist circumference (WC) (cm)**				
Normal	392 (51.9)	244 (59.5)	148 (42.8)	** < 0.001**
Abnormal	364 (48.1)	166 (40.5)	198 (57.2)	
**Fasting plasma glucose (FPG) (mg/dl)**				
Normal	484 (64)	288 (70.2)	196 (56.6)	** < 0.001**
Abnormal	272 (36)	122 (29.8)	150 (43.4)	
**Triglyceride (TG) (mg/dl)**				
Normal	498 (65.9)	242 (59)	256 (74)	** < 0.001**
Abnormal	258 (34.1)	168 (41)	90 (26)	
**High density lipoprotein-cholesterol (HDL-C) (mg/dl)**				
Normal	257 (34)	180 (43.9)	77 (22.3)	** < 0.001**
Abnormal	499 (66)	230 (56.1)	269 (77.7)	
**Blood pressure (BP) (mmHg)**				
Normal	469 (62)	206 (50.2)	263 (76)	** < 0.001**
Abnormal	287 (38)	204 (49.8)	83 (24)	
**Metabolic syndrome abnormal component**				
0 Component	89 (11.8)	57 (13.9)	32 (9.2)	**0.01**
1 Component	146 (19.3)	73 (17.8)	73 (21.1)	
2 Components	200 (26.5)	122 (29.8)	78 (22.5)	
≥ 3 Components	321 (42.5)	158 (38.5)	163 (47.1)	
**Metabolic syndrome presence**				
No	435 (57.5)	252 (61.5)	183 (52.9)	**0.018**
Yes	321 (42.5)	158 (38.5)	163 (47.1)	

Data are presented as Numbers and Percentages (N (%))

Chi-square test was performed to test the differences between genders

*P < 0.05 in the comparison between Males and Females

Significant values are in bold.

When compared to men (40.5%, 29.8%, and 56.1%, respectively), women had significantly higher abnormal values for WC (57.2%), fasting plasma glucose (FPG) level (43.4%), and HDL-C (77.7%) (*p* < 0.001). Men, on the other hand, had a significantly higher prevalence of hypertriglyceridemia (41% vs. 26%) (*p* < 0.001) and elevated blood pressure (BP) (49% vs. 24%) (*p* < 0.001) than women (Table 2).

### Anthropometric indices and MSyn

The correlation between the anthropometric indices and MSyn presence among the study population is shown in (Table 3). The mean values for BMI, WC, WHtR, ABSI, BRI, VAI, and CI, were: 28.93 kg/m^2^, 95.86 cm, 0.58, 0.08 m^11/6^ kg^−2/3^, 5.15, 6.8 cm^2^, and 1.27 m^3/2^ kg^−1/2^, respectively. Men with MSyn had significantly higher values of WC (107.8 cm), ABSI (0.08 m^11/6^ kg^−2/3^), and CI (1.37 m^3/2^ kg^−1/2^) compared to women (100.98 cm, 0.08 m^11/6^ kg^−2/3^, and 1.26 m^3/2^ kg^−1/2^, respectively) (*p* ≤ 0.001). In contrast, women with MSyn had significantly higher values of WHtR (0.64 vs. 0.63), BRI (6.59 vs. 6.29), VAI (10.37 cm^2^ vs. 9.76 cm^2^), and BMI (34.23 kg/m^2^ vs. 30.59 kg/m^2^) than did men; (*p* < 0.001).

**Table 3:** Means and standard deviations (SD) for the seven anthropometric indices among study subjects, with and without MSyn, stratified by gender.

Variable	Total Mean ± SD	Males		*P*-value	Females		*P*-value
		MSyn Presence	MSyn Absence		MSyn Presence	MSyn Absence	
Waist circumference (WC) (cm)	95.86 ± 14.58	107.8 ± 11.5	93.44 ± 11.34	** < 0.001**	100.98 ± 12.56	84.31 ± 12.65	** < 0.001**
Waist-to-height ratio (WHtR)	0.58 ± 0.09	0.63 ± 0.07	0.54 ± 0.07	** < 0.001**	0.64 ± 0.10	0.53 ± 0.05	** < 0.001**
A Body shape index (ABSI) (m^11/6^ kg^-2/3^)	0.08 ± 0.01	0.09 ± 0.01	0.08 ± 0.01	** < 0.001**	0.08 ± 0.01	0.08 ± 0.01	**0.001**
Body roundness index (BRI)	5.15 ± 2.14	6.29 ± 1.85	4.27 ± 1.53	** < 0.001**	6.59 ± 2.24	4.08 ± 1.79	** < 0.001**
Visceral adiposity index (VAI) (cm^2^)	6.8 ± 6.62	9.76 ± 8.03	4.37 ± 3.18	** < 0.001**	10.37 ± 8.95	4.41 ± 3.15	** < 0.001**
Conicity index (CI) (m^3/2^ kg^−1/2^)	1.27 ± 0.12	1.37 ± 0.1	1.28 ± 0.1	** < 0.001**	1.26 ± 0.10	1.18 ± 0.09	** < 0.001**
Body mass index (BMI) (kg/m2)	28.9 ± 6.1	30.6 ± 4.2	25.9 ± 4.5	** < 0.001**	34.2 ± 6.0	27.0 ± 5.9	** < 0.001**
**BMI classifications (N (%))**							
Underweight	12 (1.6)	0 (0)	8 (3.2)	** < 0.001**	0 (0)	4 (2.2)	** < 0.001**
Normal weight	209 (27.6)	14 (8.9)	107 (42.5)		6 (3.7)	82 (44.8)	
Overweight	244 (32.3)	57 (36.1)	103 (40.3)		36 (22.1)	48 (26.2)	
Obese	291 (38.5)	87 (55.1)	34 (13.5)		121 (74.2)	49 (26.8)	

Data are presented as Means ± SD for anthropometric indices, while presented as Numbers and Percentages (N (%)) for BMI classification.

Independent-samples t-test was performed to compare the means of anthropometric indices, while Chi-square test was performed to test the differences in the BMI classification between subjects with or without MSyn within each gender group.

*MSyn* Metabolic Syndrome.

*P < 0.05 in the comparison between subjects with or without MSyn within the same gender group.

Significant values are in bold.

A BMI ≥ 25 kg/m^2^ was seen in more than 70% of the study population, with 38.5% obese and 32.3% overweight individuals, while only about 28% of the study population had a normal body weight. Obese women and men had a significantly higher prevalence of MSyn compared to the other categories of BMI (*p* < 0.001); 74.2% of obese women and 55.1% of obese men had MSyn. This study presented twelve (1.6%) underweight participants (BMI < 18.5 kg/m^2^), and none of them were defined as having MSyn in both genders (Table 3).

### ROC curve of anthropometric indices to predict MSyn

**Figure 1:**
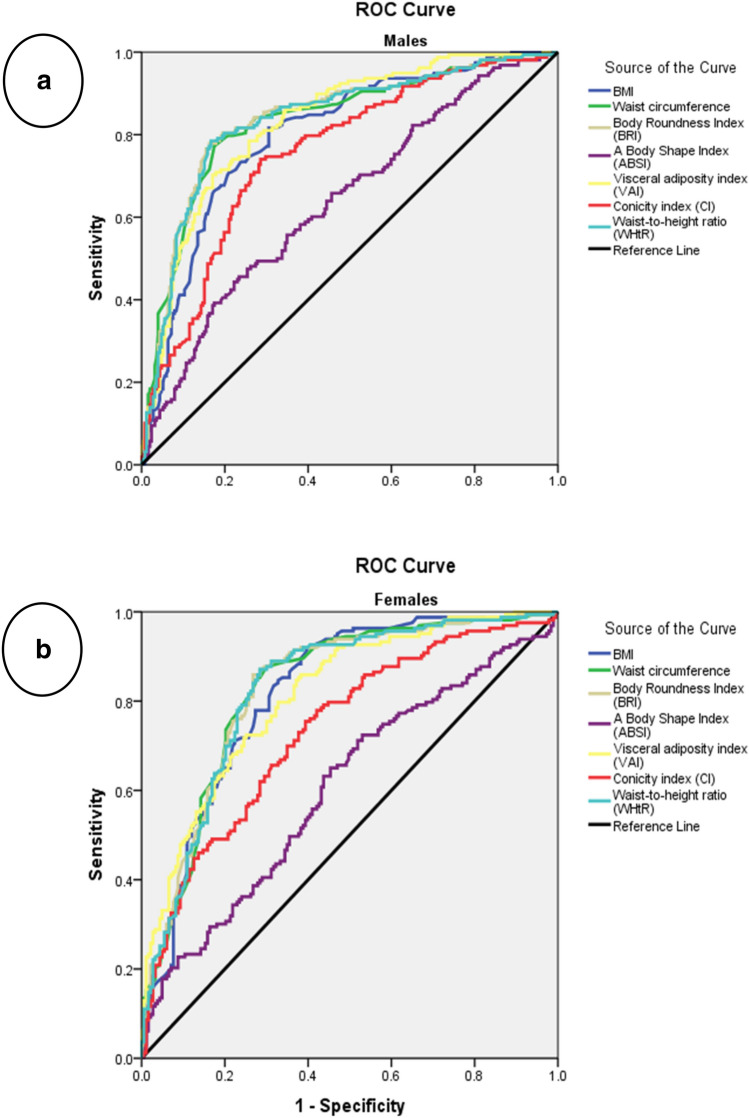
The receiver operating characteristic (ROC) curve of seven anthropometric indices describes their quality in predicting metabolic syndrome among male (**a**) and female (**b**) adults in Jordan.

The ROC curves and the area under the curve (AUC) comparing the predictive abilities of BMI, WC, BRI, ABSI, VAI, CI, and WHtR for MSyn among our study population are presented in (Table 4 and Fig. 1). For all the study population, BRI and WHtR performed equally in predicting MSyn (AUC = 0.83 for both). Similarly, in men, BRI and WHtR performed equally in predicting MSyn (AUC = 0.84 for both). For women, WC, BRI, and WHtR performed equally in predicting MSyn (AUC = 0.83 for all). BRI and WHtR had a superior ability to predict MSyn presence than the other indices.

**Table 4:** Area under ROC curve (AUC), optimal cutoff values, sensitivities, specificities, and Youden index of anthropometric indices in predicting MSyn.

Variables	AUC (95% CIs)	Cutoff	Sensitivity (95% CIs)	Specificity (95%CIs)	Youden index
**Total**					
BMI	0.81 (0.78–0.84)	> 28.4	77.88	73.79	0.5167
WC	0.80 (0.77–0.83)	> 98.5	71.03	78.16	0.4919
BRI	0.83 (0.80–0.86)	> 5.133	77.57	79.77	0.5734
ABSI	0.58 (0.54–0.62)	> 0.0853	24.92	88.97	0.1389
VAI	0.82 (0.79–0.85)	> 5.55231	71.34	77.7	0.4904
CI	0.69 (0.65–0.73)	> 1.325	47.98	81.38	0.2935
WHtR	0.83 (0.80–0.86)	> 0.586	77.88	78.85	0.5673
**Males**					
BMI	0.80 (0.76–0.84)	> 27.4	81.65	69.44	0.5109
WC	0.83 (0.79–0.87)	> 102	77.22	82.54	0.5975
BRI	0.84 (0.80–0.87)	> 5.296	77.85	83.73	0.6158
ABSI	0.64 (0.59–0.69)	> 0.0843	45.75	76.59	0.2216
VAI	0.83 (0.79–0.87)	> 6.2842	70.25	82.94	0.5319
CI	0.76 (0.71–0.8)	> 1.325	74.05	71.43	0.4548
WHtR	0.84 (0.80–0.87)	> 0.594	78.48	83.33	0.6181
**Females**					
BMI	0.82 (0.77–0.86)	> 26.8	92.02	85.2	0.5213
WC	0.83 (0.78–0.87)	> 88	87.73	69.95	0.5768
BRI	0.83 (0.79–0.87)	> 4.695	85.89	73.22	0.5911
ABSI	0.60 (0.54–0.66)	> 0.07484	65.03	54.64	0.1968
VAI	0.81 (0.77–0.86)	> 4.04102	85.89	61.75	0.4764
CI	0.73 (0.68–0.79)	> 1.193	75.46	60.66	0.3612
WHtR	0.83 (0.78–0.87)	> 0.565	81.12	71.04	0.5815

Discriminatory power of the anthropometric indices as classifiers for MSyn presence was determined by ROC curve. AUCs and the 95%CI were used to compare the discriminatory power of each index. Sensitivity was defined as the percentage of true positive scores according to the MSyn presence. Specificity was defined as the proportion of scores identified incorrectly. Optimal cutoff points for all seven obesity indicators were determined with Youden's J (J max. = Sensitivity + Specificity– 1)

*ROC* Receiver operative characteristic curve, *AUC* area under the curve, *CIs* confidence intervals, *MSyn* Metabolic Syndrome, *BMI* body mass index, *WC* waist circumference, *BRI* body roundness index, *ABSI* a body shape index, *VAI* Visceral Adiposity Index, *CI* conicity index, *WHtR* Waist-to-height ratio.

In the total population, regardless of gender, BMI and WHtR showed the highest sensitivity level of 77.88% in detecting MSyn. The results showed that females had a higher sensitivity level of BMI (92.02% vs. 81.65%), WC (87.73% vs. 77.22%), BRI (85.89% vs. 77.85%), ABSI (65.03% vs. 45.75%), VAI (85.89% vs. 70.25%), CI (75.46% vs. 74.05%), and WHtR (81.12% vs. 78.48%) than males. Moreover, the AUCs of BMI, WC, BRI, VAI, and WHtR showed good discrimination ability, while both ABSI and CI failed to discriminate MSyn presence.

For the study population, the optimal cutoff point for an early diagnosis of MSyn was > 28.4 kg/m^2^ for BMI, > 98.5 cm for WC, > 5.13 for BRI, > 0.09 m^11/6^ kg^-2/3^ for ABSI, > 5.55 cm^2^for AVI, > 1.33 m^3/2^ kg^−1/2^ for CI, and > 0.59 for WHtR. Employing gender differences, males have higher anthropometric indices cutoff points for MSyn early detection as follows: BMI (> 27.4 kg/m^2^ vs. > 26.8 kg/m^2^), WC (> 102 cm vs. > 88 cm), BRI (> 5.296 vs. > 4.695), ABSI (> 0.084 m^11/6^ kg^-2/3^ vs. > 0.075 m^11/6^ kg^-2/3^), VAI (> 6.284 cm^2^ vs. > 4.041 cm^2^), CI (> 1.325 m^3/2^ kg^−1/2^ vs. > 1.193 m^3/2^ kg^−1/2^), and WHtR (> 0.594 vs. > 0.565) compared to females. Meanwhile, the specificity and Youden index of these obesity-related anthropometric measurements that predicted MSyn are also presented in (Table 3). The results showed that the studied anthropometric indices had a significant predictive ability for MSyn in each gender and the whole study population (*P* < 0.0001) (Table 4 and Fig. 1).

## Discussion

Five risk factors were commonly used to define MSyn including elevated WC, elevated FPG or previously diagnosed Type 2 DM, elevated TG, reduced HDL-C, elevated BP, or having a specific treatment for these abnormalities^34,35^. In addition, other lifestyle factors were well recognized as critical modifiable risk factors for MSyn such as smoking^35,36^. In this study, a higher tendency for smoking was found in males than females, which was consistent with previous studies among Jordanian adults^37^. Nevertheless, MSyn prevalence was higher in females than males in this study which might be affected by other factors or might be related to not taking hookah smoking into consideration. Hookah smoking recently became more socially accepted among Jordanian adults, particularly among females^38^. A study among Iranian women showed that smoking is not a predictor for MSyn, besides, the study presents a higher prevalence of MSyn among non-smoker women compared to smoker women^39^. In comparison, a cohort study among adults in Saudi Arabia found that smoking was independently and significantly associated with an increased risk of MSyn (Odd Ratio = 1.4)^40^. In Korea, smoker young adults were 2.4 times more likely to be diagnosed with MSyn than non-smokers, and they were more likely to have MSyn components^41^. Another study in Puerto Rico showed that the association between smoking and the risk of having MSyn might depend on smoking heaviness. Also, it showed a higher prevalence of MSyn and some of its components among former smokers than current or never smokers^42^. However, due to the considerable prevalence of smoking, 33% of the population in this study, future studies are needed to emphasize the role of different types of smoking as a determinant factor for MSyn and its components among Jordanian adults.

Using the Adult Treatment Panel III (ATP III) definition, a considerable prevalence of individual metabolic abnormalities was identified among our study population; the prevalence of these metabolic abnormalities was close to the results published among adults in Jordan and Iran^43,44^. The prevalence of a single MSyn component might differ between men and women. The current study presented women with a higher incidence of metabolic abnormalities including elevated FPG, and low HDL-C. In contrast, Ajlouni and colleagues^43^ showed that men had a higher prevalence of elevated FPG, and low HDL-C, although the difference was not significant. It is noteworthy to mention that our results were consistent with Ajlouni and colleagues^43^ study as men had a higher prevalence of hypertriglyceridemia and high blood pressure when compared to women. In other neighboring Arab countries such as Saudi Arabia, the prevalence of MSyn risk factors including elevated TG, BP, and FPG was higher in men except for the low HDL-C levels that occurred more in women^45^. Also, a study among Arab and South Asian Residents in Kuwait presented men with more incidence of metabolic abnormalities including hypertension, elevated FPG, and TG in both Arabs and South Asian groups. Abnormal low levels of HDL-C, was seen in Arab men more than Arab women, but it was higher among Asian women than men^46^. In the East of Asia, specifically China, more women had an abnormal WC, HDL-C, TC, and LDL-C compared to men, whereas hypertension, elevated TG and FPG levels occurred in men more than in women^47^. The prevalence of abnormal WC among our participants was 48%, which was in line with previously published results of abnormal WC using the Adult Treatment Panel III (ATP III) definition (56.5%), but low compared to the results while using the International Diabetes Federation (IDF) criteria for an abnormal WC (74.6%)^43^. The study found that more females had an abnormal WC compared to males. The results were consistent with a previous study among Jordanian adults as 67.3% of males vs. 77.8% of females had abnormal WC according to the IDF criteria. Referring to the ATP III definition 62.8% of females and 41.5% of males had an abnormal WC, which is similar to the prevalence of abnormal WC among our population^43^.

Our results were consistent with a previous study among Jordanian adults as women were more likely to have MSyn than men with an OR of 1.42^43^. Likewise, in Iran, a middle eastern country, MSyn prevalence was 3.2 times higher among females than males^44^. MSyn prevalence in an individual population might differ according to the diagnostic criteria used in the study. Even though all MSyn definitions share the same metabolic components, the differences in the WC cutoff points and the necessity of its inclusion in the diagnostic criteria of MSyn might result in a different prevalence^48^. MSyn was found in 42.5% of our study population, which is nearly the same as found by Ajlouni and colleagues^43^ who noticed a higher prevalence of MSyn among Jordanian adults using the IDF criteria compared to ATP III criteria (48.2% vs. 44.1%), although the results showed an excellent agreement between the definitions. As well, more men met MSyn criteria compared to women using both IDF (52.9% vs. 46.2%) and ATP III (51.4% vs. 41.0%) definitions^43^. Similarly, in Iran the prevalence of MSyn was higher using the IDF criteria compared to ATP III criteria (42.9% vs. 40.7%) although the results were quiet close to each other^44^. In comparison, more Saudi adults were diagnosed with MSyn using ATP III compared to the IDF definition (39.8% vs. 31.6%). However, the prevalence of MSyn was higher in men than women in both cases^40^. In Kuwait, it was found that more men were diagnosed with MSyn compared to women in Arab and South Asian residents, irrespective of the WC cutoff or MSyn criteria used in the study^46^. Previous studies in two different countries in East Asia showed a different sex-specific pattern of MSyn prevalence. Chinese women had a higher prevalence of MSyn than men (57.9% vs 38.4%), while in Korean men had a higher prevalence of MSyn than women (4.63% vs. 31.91%)^41,47^. Cho and colleagues^49^ presented Korean men with a higher prevalence of MSyn (65.7% vs. 55.7%), and also a higher incidence of individual metabolic abnormality compared to Korean women. In Mexico, in North America, women were having MSyn more than men (36.0% vs. 11.2%)^50^.

Anthropometry has been widely used as a noninvasive assessment tool to indicate health risks or to identify an individual's nutritional status^51^. The individual's measurements are usually compared with the reference values for the same age and sex groups related to a population from the same ethnic origin. For some populations, obtaining reference data might be challenging which obligates them to use the international reference values^52^. However, the anthropometric measurements might indicate different health risks in different racial groups. In Kuwait, it was found that Arabs had a higher mean of WC and BMI, yet, they had a lower percentage of metabolic abnormalities including hypertension, diabetes, dyslipidemia, and a lower prevalence of three or more CVD risk factors when compared to south Asians who reside in the same country^46^. Thus, the development of anthropometric indicators and cutoff points that will be appropriate for specific subgroups of race, age, and sex was recommended by the WHO^53^. Referring to the report of the WHO, the cutoff points for WC associated with increased risk of metabolic complications among some populations in the middle east were similar to those of the European populations (94 cm for men and 80 cm for women)^54^. These cutoffs were also determined by the IDF for Europids men and women, and South Asians, Chinese and Japanese women. Just for South Asians, Chinese and Japanese men the determined WC cutoff value was 90 cm^55^. In this study, the WC cutoffs for early prediction of MSyn were > 102 cm for men and > 88 cm for women; which is higher than those recommended by the WHO for the middle east population but the same as the values published by National Cholesterol Education Program^56^. As the appearance of MSyn phenotype is triggered by intra-abdominal fat accumulation which is mirrored by a large WC^57^, applying the newly established cutoff points in MSyn diagnosis might result in a better prediction for the syndrome. For example, in Egypt applying the suggested WC cutoff values in the Joint Interim Statement (JIS) definition of the metabolic syndrome might be the most suitable to predict MSyn among Egyptian adults^58^. Another study conducted in Kuwait found that the use of IDF WC cutoffs corresponded to a higher prevalence across sex and ethnic groups compared with the sample suggested cutoffs, which might lead to overestimation of MSyn prevalence^46^.

Our study showed that males had higher cutoff points for all the anthropometric indices than females. The results were consistent with studies from countries in Europe and East Asia, except for BRI as Polish females had a higher cutoff value than males^49,59^. In Spain, males and females had the same cutoffs for ABSI and WHtR^59^. In some populations, such as Saudi adults, females had a higher cutoff value for BMI and WC in MSyn prediction. (Supplementary Table 1) presents some of the anthropometric indices and their cutoff values in different populations. In this study, BRI and WHtR performed equally in predicting MSyn among adult men, while in women, WC, BRI, and WHtR performed equally in predicting MSyn. In Egypt and Saudi Arabia, nearby Arabic countries, WC was the best index to predict MSyn in both men and women^58^. In East Asia, WHtR was among the best predictors of MSyn. For example, in China, WHtR and BRI were the best predictors in diabetic men and women aged between 40–59 years, while in adults older than 60 years other indices including BMI in women and Clínica Universidad de Navarra-body adiposity estimator (CUN-BAE) in men had the highest MSyn discrimination ability^26^. Another example, in Korea, WHtR was the best predictor for MSyn in men and women while the CI had the lowest prediction power^49^. A comparison between Arabs and South Asian adults residing in Kuwait showed that BMI was the best predictor for MSyn in Asian women, and WHtR was the best predictor in Asian men. But, for Arabs BMI was the index with the highest prediction ability in men, and both BMI and WHtR predicted MSyn equally among Arab women^46^. In two European countries, Poland and Spain, the ABSI was the anthropometric index that had the lowest discrimination power for predicting MSyn in men and women^59,60^, which was consistent with the results of the current study as ABSI had the lowest AUC and it was the only index that failed to discriminate MSyn risk. Both WHtR and BMI were among best MSyn predictors in Spanish women with an intermediate risk for CVDs, while BMI was marked as the best predictor in men^59^. Also, in Polish women WHtR had the largest discrimination ability, while in men the best prediction was for CUN-BAE, followed by BMI, and WC^60^.

The results of the current study might be limited by the use of cross-sectional design, as the temporal association between the studied variables and disease outcome, in addition to the causality cannot be determined. Also, using the ROC curves to figure out the population-specific cutoff values are used in different countries, however, the concern is that these cutoff points might differ based on differences in population characteristics such as the disease prevalence in the studied population^54^. Also, using the AUC ROC method might result in False-positive and false-negative diagnoses that have different misclassification costs. Further, there is a chance of an excessive ROC curve extrapolation which is detrimental^61^. However, to the best of our knowledge, this is the first study that examines the ability of seven anthropometric indices to predict MSyn prevalence among Jordanian adults. These results might be helpful for the health sector in Jordan to select the best index such as WHtR or BRI for MSyn prediction rather than merely relying on the traditionally used measurements of BMI and WC as the newly suggested indices are affordable and might result in better MSyn prediction. There are other contributing factors to the development of MSyn that can be studied in the future in their relation to the anthropometric indices such as the dietary and lifestyle factors.

## Methods

### Subjects selection and study design

#### Ethical considerations

The sample size was calculated with a 5% error and 99% confidence level based on the Jordanian Department of Statistics’ latest census reports^62^. The minimum sample needed was 650 subjects; 800 subjects were invited to participate in this study in order to increase the study power, of which 756 subjects were enrolled and agreed to participate in the study. Before enrolling in the study, each willing and eligible subject to participate provided informed written consent. No incentives were offered for taking part in the study.

The research was performed following the Declaration of Helsinki, and the study was approved by the Research and Ethical Committee at the Jordan University of Science and Technology (IRB approval reference number 16/115/2018).

Using a standardized questionnaire and the participant's consent, a face-to-face interview was carried out to collect sociodemographic information. There were questions about the presence of chronic diseases and other health problems as well as smoking status. An appointment was made to gather the participants' biochemical information after the interviews, while anthropometrics and blood pressure were measured and recorded during the interviews.

### Sample and sampling technique

A population-based cross-sectional study was conducted in the Hashemite Kingdom of Jordan between May 2018 and September 2019. It was carried out among apparently healthy adults aged more than 20 years to determine the prevalence of non-communicable diseases and the associated risk factors. The target sample was drawn from governmental and military hospitals in Jordan's north, middle, and southern provinces and was collected randomly from these hospitals' outpatient clinics. Patients from both genders who regularly visited these hospitals for follow-up care and/or their caregivers, and who were > 20 years of age were invited to participate in the study.

### Inclusion and exclusion criteria

For the non-communicable diseases screening in this study, subjects were randomly selected regardless of their disease state, with patients with diabetes, hypertension, and dyslipidemia included. Exclusion criteria included female subjects who were pregnant or lactating, non-Jordanian subjects, subjects with mental disorders, and any terminally ill subjects. In addition, subjects with incomplete anthropometric measurements and/or biochemical parameters were also excluded.
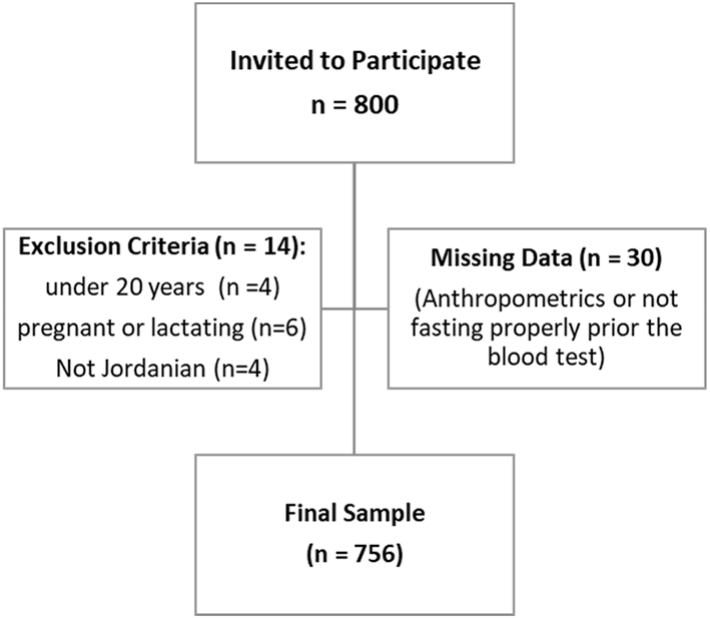


### Anthropometric measurements

Height was measured by portable stadiometers with the participants' feet placed together with heels, buttocks, and shoulder blades against the stick and head positioned in the Frankfurt horizontal plane; height was measured to the nearest 0.5 cm. Weight was measured with a portable electronic scale which was calibrated according to the manual directions. The scale was placed on a hard flat surface and checked for zero balance before taking each measurement. Subjects were weighed standing on their feet, barefooted, and wearing light clothes; their weight was recorded to the nearest 0.1 kg. Waist circumferences were measured using a non-elastic measuring tape (Seca), it was measured at the narrowest level between the lowest rib and the iliac crest at the end of normal expiration, and measurements were recorded to the nearest 0.1 cm.

### Anthropometric indices calculations

Six anthropometric indices were calculated, including BMI, WHtR, ABSI, BRI, CI, and VAI. The calculation formulas used in this study are shown below:

- BMI= $$Weight (kg)/({\left(Height \left(m\right)\right)}^{2}$$ ^63^
- WHtR= $$\frac{Waist circumference (cm)}{Height (cm)}$$ ^64^
- ABSI = $$Waist circumference (m)/ (({Height (m))}^{0.5}*\left({BMI}^\frac{2}{3}\right))$$ ^13^
- BRI=$$364.2-365.5\sqrt{\begin{array}{c}1-\left(\frac{{\left(Waist circumference(cm)/2\pi \right)}^{2}}{{\left(0.5*Height \left(cm\right)\right)}^{2}}\right)\\ \end{array}}$$ ^16^
- CI = $$Waist circumference (m)/(0.109*\sqrt{\begin{array}{c}\left(\frac{Weight\left(kg\right)}{height\left(m\right)}\right)\end{array}}$$) ^19^
- VAI male = [WC (cm)/39.68 − 1.88BMI (kg/m^2^)] [TG (mmol/L)/1.03] [1.31/HDL (mmol/L)] ^21^
- VAI female = [WC (cm)/36.58 − 1.89BMI (kg/m^2^)] [TG (mmol/L)/0.81] [1.52/HDL (mmol/L)]. ^21^

### Blood pressure measurement

A standard oscillometric sphygmomanometer (type USM- 700GSi, Elquest Corporation, Chiba, Japan) was used to measure the blood pressure of each subject in the sitting position after a 5-min rest period by a trained, qualified nurse. The blood pressure was measured in the right arm two times, and the average of two measurements was used for data analysis.

### Serum biochemical examination

A 10 mL of blood was obtained from all individuals after 12 h of overnight fast. All samples were centrifuged. Serum samples were subsequently analyzed for measuring fasting plasma glucose (FPG), total cholesterol (TC), triglyceride (TG), and HDL-cholesterol (HDL-C) by a compact clinical chemistry analyzer (Hitachi 902 auto-analyzer, Roche; Germany). The Roche/Hitachi 902 principle uses colorimetry and absorbance measurement via the ion-selective electrode method to analyze serum samples.

### MSyn definition

According to the National Cholesterol Education Program (NCEP), Third Adult Treatment Panel III (ATP III) criteria criteria, the diagnosis of MSyn was made when three or more of the following risk factors are present: a waist circumference (WC) ≥ 102 cm (≥ 40 inches) in men and ≥ 88 cm (≥ 35 inches) in women, fasting plasma glucose (FPG) ≥ 100 mg/dl (5.55 mmol/l) or on drug treatment for elevated glucose, systolic blood pressure (SBP) ≥ 130 mmHg or diastolic blood pressure (DBP) ≥ 85 mmHg or on antihypertensive drug treatment in a patient with a history of hypertension, fasting triglycerides (TG) ≥ 150 mg/dl (1.7 mmol/l) or on drug treatment for elevated TG, and HDL-C ≤ 40 mg/dl (1.0 mmol/l) in men and ≤ 50 mg/dl (1.3 mmol/l) in women or on drug treatment for reduced HDL-C^65^.

### Statistical analysis

Data were analyzed using the IBM SPSS Statistics (IBM Corp. Released 2012. IBM SPSS Statistics for Windows, Version 22.0. Armonk, NY: IBM Corp). Chi-square (χ2) test was used for categorical variables which were presented as numbers and percentages (n (%)). Continuous variables were tested using an Independent-samples t-test and presented as means ± SD. P-values < 0.05 were considered statistically significant. Missing data were treated using stepwise deletion method, wherein, all the cases with missing values were omitted from the analysis. The Shapiro–wilk test was applied to assess the normality of the sample. Receiver-operating characteristic (ROC) curve was used to determine the discriminatory power of the anthropometric indices as classifiers for MSyn presence.

The areas under the curve (AUCs) and the 95% confidence intervals (CI) were used to present the ROC result. Sensitivity was defined as the percentage of true positive scores according to the presence of MSyn defined using the ATP III criteria. Specificity was defined as the proportion of scores identified incorrectly. The AUC measures the precision of a given index in the differentiation between individuals with MSyn and without it. It also characterizes the probability of assigning a patient to the correct group. For the AUC, 0.5 was adopted as the bottom borderline. The indices with the biggest AUC were considered the best. Optimal cutoff points for all seven obesity indicators were determined with Youden's J statistic using the following equation: Jmax. = Sensitivity + Specificity– 1. The index values corresponding to the maximum value of Youden's J statistic were recognized as optimal cutoff points for these indices. An AUC between 0.9 and 1.0, with 95%CI, showed an excellent MSyn discrimination ability using the index. AUC from 0.80 to 0.90 indicates good discrimination. However, lower AUC values ranging from 0.70 to 0.80, 0.60 to 0.70, and 0.50 to 0.60 were considered fair, poor, and failed indices for predicting MSyn, respectively^66^.

## Supplementary Information

Supplementary Information 1.

Supplementary Information 2.

## Data Availability

Data available on request due to restrictions (privacy or ethical); contact the corresponding author.
