# Supplementary material for: Assessing metabolic syndrome prediction quality using seven anthropometric indices among Jordanian adults: a cross-sectional study
Source: Sci Rep. 2022 Dec 6;12:21043. doi: 10.1038/s41598-022-25005-8 (PMC9727133; doi:10.1038/s41598-022-25005-8)
Supplement: Supplementary file 1 — Supplementary Information 1. [file 41598_2022_25005_MOESM1_ESM.doc]

STROBE Statement—Checklist of items that should be included in reports of ***cross-sectional studies***

|  | Item No | Recommendation | Notes |
| --- | --- | --- | --- |
| **Title and abstract** | 1 | (*a*) Indicate the study’s design with a commonly used term in the title or the abstract | Line 2 |
|  |  | (*b*) Provide in the abstract an informative and balanced summary of what was done and what was found | Line 37-46 |
| Introduction | | |  |
| Background/rationale | 2 | Explain the scientific background and rationale for the investigation being reported | Line 103-117 |
| Objectives | 3 | State specific objectives, including any prespecified hypotheses | Line 110-117 |
| Methods | | |  |
| Study design | 4 | Present key elements of study design early in the paper | Line 369 – 370 |
| Setting | 5 | Describe the setting, locations, and relevant dates, including periods of recruitment, exposure, follow-up, and data collection | Line 370-376 |
| Participants | 6 | (*a*) Give the eligibility criteria, and the sources and methods of selection of participants | Line 348-351  Line 379-385 |
| Variables | 7 | Clearly define all outcomes, exposures, predictors, potential confounders, and effect modifiers. Give diagnostic criteria, if applicable | Line 389-399  Line 412-425  Line 426-438 |
| Data sources/ measurement | 8* | For each variable of interest, give sources of data and details of methods of assessment (measurement). Describe comparability of assessment methods if there is more than one group | Anthro (389-399). Formulas (400-411).  BP:412-417)  Biochemical (418-425) |
| Bias | 9 | Describe any efforts to address potential sources of bias | Line 326-334 |
| Study size | 10 | Explain how the study size was arrived at | Line 347 – 351 |
| Quantitative variables | 11 | Explain how quantitative variables were handled in the analyses. If applicable, describe which groupings were chosen and why | Line 443-444 |
| Statistical methods | 12 | (*a*) Describe all statistical methods, including those used to control for confounding | NA |
|  |  | (*b*) Describe any methods used to examine subgroups and interactions | Line 445 – 447 |
|  |  | (*c*) Explain how missing data were addressed | Line 444 – 445 |
|  |  | (*d*) If applicable, describe analytical methods taking account of sampling strategy | Line 347 – 351 |
|  |  | (*e*) Describe any sensitivity analyses | Line 449-452 |
| Results | | |  |
| Participants | 13* | (a) Report numbers of individuals at each stage of study—eg numbers potentially eligible, examined for eligibility, confirmed eligible, included in the study, completing follow-up, and analysed | Line 379-386  (Flow diagram) |
|  |  | (b) Give reasons for non-participation at each stage | Flow diagram, Line 349-351 |
|  |  | (c) Consider use of a flow diagram | Page 17 |
| Descriptive data | 14* | (a) Give characteristics of study participants (eg demographic, clinical, social) and information on exposures and potential confounders | Line 121 – 123, Table 1 |
|  |  | (b) Indicate number of participants with missing data for each variable of interest | NA |
| Outcome data | 15* | Report numbers of outcome events or summary measures | O1 (127– 137)  O2 (138 – 155)  O3 (156 – 183) |
| Main results | 16 | (*a*) Give unadjusted estimates and, if applicable, confounder-adjusted estimates and their precision (eg, 95% confidence interval). Make clear which confounders were adjusted for and why they were included | Table 4 |
|  |  | (*b*) Report category boundaries when continuous variables were categorized | Table 1 (Age)  Table 2 (Metabolic abnormalities)  Line 441 – 444 |
|  |  | (*c*) If relevant, consider translating estimates of relative risk into absolute risk for a meaningful time period | NA |
| Other analyses | 17 | Report other analyses done—eg analyses of subgroups and interactions, and sensitivity analyses | Line 441 – 452 |
| Discussion | | |  |
| Key results | 18 | Summarise key results with reference to study objectives | Line 297-325 |
| Limitations | 19 | Discuss limitations of the study, taking into account sources of potential bias or imprecision. Discuss both direction and magnitude of any potential bias | Line 326 – 334 |
| Interpretation | 20 | Give a cautious overall interpretation of results considering objectives, limitations, multiplicity of analyses, results from similar studies, and other relevant evidence | Line 331 – 334 |
| Generalisability | 21 | Discuss the generalisability (external validity) of the study results | NA |
| Other information | | |  |
| Funding | 22 | Give the source of funding and the role of the funders for the present study and, if applicable, for the original study on which the present article is based | Line 673-674 |

*Give information separately for exposed and unexposed groups.

**Note:** An Explanation and Elaboration article discusses each checklist item and gives methodological background and published examples of transparent reporting. The STROBE checklist is best used in conjunction with this article (freely available on the Web sites of PLoS Medicine at http://www.plosmedicine.org/, Annals of Internal Medicine at http://www.annals.org/, and Epidemiology at http://www.epidem.com/). Information on the STROBE Initiative is available at www.strobe-statement.org.
