# Supplementary material for: Assessing metabolic syndrome prediction quality using seven anthropometric indices among Jordanian adults: a cross-sectional study
Source: Sci Rep. 2022 Dec 6;12:21043. doi: 10.1038/s41598-022-25005-8 (PMC9727133; doi:10.1038/s41598-022-25005-8)
Supplement: Supplementary file 2 — Supplementary Information 2. [file 41598_2022_25005_MOESM2_ESM.docx]

Assessing Metabolic Syndrome Prediction Quality Using Seven Anthropometric Indices among Jordanian adults: A Cross-Sectional Study

Islam Al-Shami^1^, Hana Alkhalidy^2,*^, Khadeejah Alnaser^2^, Tareq L Mukattash^3^, Huda Al Hourani^1^, Tamara Alzboun^2^, Aliaa Orabi^2^, Dongmin Liu^4^

| **Supplementary Table S1.** Optimal cutoff values of anthropometric indices in predicting metabolic abnormalities in different populations | | | | | | | | | | | | | | | | | |
| --- | --- | --- | --- | --- | --- | --- | --- | --- | --- | --- | --- | --- | --- | --- | --- | --- | --- |
| **Country** | | **Data collection (year)** | **Index** | | | | | | | | | | | | | | |
|  |  |  | **BMI (kg/m^2^)** | | **WC (cm)** | | **BRI** | | **ABSI**  **(m^11/6^ kg^-2/3^)** | | **VAI (cm^2^)** | | **CI (m^3/2^/kg^1/2^)** | | **WHtR (cm/cm)** | | **Ref.** |
|  |  |  | M | F | M | F | M | F | M | F | M | F | M | F | M | F |  |
| **Jordan** | | (2018-2019) | 27.4 | 26.8 | 102 | 88 | 5.296 | 4.695 | 0.084 | 0.075 | 6.284 | 4.041 | 1.325 | 1.193 | 0.594 | 0.565 | Current |
| **Poland** | | (2010–2012) | 27.65 | 27.41 | 94.0 | 88.0 | 4.612 | 4.934 | 0.081 | 0.076 | - | - | - | - | 0.549 | 0.532 | [^1^](#_ENREF_1) |
| **Spain** | | (2011-2013) | 28.96 | 28.02 | - | - | 5.69 | 5.77 | 0.08 | 0.08 | - | - | - | - | 0.61 | 0.61 | [^2^](#_ENREF_2) |
| **Korea** | | (2004-2013) | 24.3 | 23.4 | 84.4 | 77.0 | - | - | - | - | - | - | 1.2 | 1.18 | 0.499 | 0.496 | [^3^](#_ENREF_3) |
| **Kuwait** | **Arab** | (2011-2014) | 28 | 30 | 97 | 93 | - | - | - | - | - | - | - | - | 0.55 | 0.60 | [^4^](#_ENREF_4) |
| **Kuwait** | **Asian** | (2011-2014) | 25 | 32 | 91 | 93 | - | - | - | - | - | - | - | - | 0.52 | 0.49 | [^4^](#_ENREF_4) |
| **Saudi Arabia** | | (2007-2008) | 25 | 28 | 92 | 87 | - | - | - | - | - | - | - | - | - | - | [^5^](#_ENREF_5) |
| **Mexico** | | (2018) | - | - | 100.5 | 85.5 | - | - | - | - | - | - | - | - | - | - | [^6^](#_ENREF_6) |
| **Egypt** | | (2009-2011) | - | - | 100.5 | 96.25 | - | - | - | - | - | - | - | - | - | - | [^7^](#_ENREF_7) |
| M: Males, F: Females. Data were derived from previously published studies.  BMI: body mass index; WC: waist circumference; BRI: body roundness index; ABSI: a body shape index; VAI: Visceral Adiposity Index; CI: conicity index; WHtR: Waist-to-height ratio. | | | | | | | | | | | | | | | | | |

**supplemenTary material**

**References**

1 Głuszek, S. *et al.* Anthropometric indices and cut-off points in the diagnosis of metabolic disorders. *PloS one* **15**, e0235121 (2020).

2 Gomez-Marcos, M. A. *et al.* Capacity adiposity indices to identify metabolic syndrome in subjects with intermediate cardiovascular risk (MARK study). *PLoS One* **14**, e0209992 (2019).

3 Cho, S. *et al.* Optimal cutoff values for anthropometric indices of obesity as discriminators of metabolic abnormalities in Korea: results from a Health Examinees study. *BMC Public Health* **21**, 1-8 (2021).

4 Oguoma, V. M. *et al.* Anthropometric cut-points for discriminating diabetes and the metabolic syndrome among Arabs and Asians: the Kuwait Diabetes Epidemiology Program. *British Journal of Nutrition* **127**, 92-102 (2022).

5 Al-Rubean, K. *et al.* Anthropometric cutoff values for predicting metabolic syndrome in a Saudi community: from the SAUDI-DM study. *Annals of Saudi medicine* **37**, 21-30 (2017).

6 Banik, S. D. *et al.* Evaluation of anthropometric indices and lipid parameters to predict metabolic syndrome among adults in Mexico. *Diabetes, Metabolic Syndrome and Obesity: Targets and Therapy* **14**, 691 (2021).

7 Assaad-Khalil, S. H. *et al.* Optimal waist circumference cutoff points for the determination of abdominal obesity and detection of cardiovascular risk factors among adult Egyptian population. *Indian journal of endocrinology and metabolism* **19**, 804 (2015).
